# *‘You’re a human being and not a robot that goes out to work’*: A qualitative study exploring factors impacting on wellbeing and intention to leave among lone working healthcare assistants providing palliative and end-of-life care in the community

**DOI:** 10.1177/02692163261426184

**Published:** 2026-03-18

**Authors:** Katarzyna A. Patynowska, Tracey McConnell, Anne Finucane, Emma Maun, Erin Raquel Fantoni, Jonathan Clemo, Epiphany Leone, Natasha Wynne, Felicity Hasson

**Affiliations:** 1 Marie Curie, Belfast, UK; 2 Institute of Nursing and Health Research, Ulster University, Belfast, UK; 3 School of Nursing and Midwifery, Queen’s University Belfast, Medical Biology Centre, UK; 4 Marie Curie, London, UK; 5 Clinical Psychology, Health in Social Science, University of Edinburgh, UK; 6 Marie Curie, Edinburgh, UK; 7 Marie Curie Palliative Care Research Department, University College London, UK

**Keywords:** palliative care, nursing assistants, health services research, personnel turnover, occupational stress

## Abstract

**Background:**

Healthcare assistants (paid nursing support workers without professional qualifications) provide care for terminally ill patients but remain understudied in palliative care research. Working alone in patients’ homes presents a distinct set of intersecting challenges: decision-making in distressing situations, professional isolation and limited access to training and support. Despite growing recognition of the importance of staff wellbeing for care quality and workforce retention, little is known about factors affecting wellbeing and intention to leave among this workforce.

**Aim:**

To explore factors affecting wellbeing and intention to leave among lone working healthcare assistants providing palliative and end-of-life care in the community.

**Design:**

Qualitative study combining free-text survey responses and semi-structured interviews, analysed using reflexive thematic analysis.

**Setting/participants:**

Healthcare assistants from a charitable hospice organisation in the United Kingdom.

**Results:**

One-hundred and eighty-three participants completed free-text survey questions; 14 participated in interviews. A number of societal, organisational and individual factors impacting on wellbeing and intention to leave were generated. Healthcare assistants experience high job satisfaction from meaningful work alongside systematic undervaluation. Many factors that influence wellbeing and intention to leave fall within organisational control, particularly support systems, workplace culture, recognition, and training opportunities.

**Conclusions:**

Organisations have clear opportunities to improve healthcare assistants’ wellbeing and retention through targeted interventions addressing support, recognition, and professional development needs.

**What is already known about the topic?**

- Staff wellbeing affects care quality and likelihood of leaving healthcare roles.
- Healthcare assistants face unique challenges providing palliative and end-of-life care in the community.

**What this paper adds?**

- Factors affecting lone working healthcare assistants’ wellbeing and intention to leave operate at three distinct levels: societal, organisational, and individual.
- High job satisfaction from meaningful palliative and end-of-life care coexists with systemic undervaluation of the healthcare assistants’ role, raising concerns that intrinsic motivation may compensate for structural shortfalls.
- Support systems, recognition, and professional development opportunities represent key organisational levers for improving healthcare assistants’ wellbeing and reducing intention to leave.

**Implications for practice, theory or policy**

- Future research should develop a theoretical understanding of what works, for whom, and in what circumstances to improve the wellbeing of lone working healthcare assistants providing palliative and end-of-life care in the community and reduce intention to leave.
- Investment in workplace culture and recognition systems is essential.

## Background

The wellbeing of healthcare staff has become a prominent research focus and a pressing concern for healthcare organisations.^1–4^ This attention stems in part from growing evidence linking staff wellbeing to the safety and quality of care provided^5–10^ and the likelihood of workers leaving their roles.^11,12^ In recent years, issues related to staff turnover have become more pronounced, particularly in the aftermath of the COVID-19 pandemic.^13^ Despite extensive research on the wellbeing of healthcare professionals, there remains a significant gap in the literature concerning healthcare assistants. Yet, this workforce plays a vital role across many countries in delivery of direct care, such as providing personal care, monitoring changes that require escalation to other professionals, emotional support for terminally ill patients and their families.^14–17^

Internationally, healthcare assistants are recognised as support healthcare workers and often operate without the oversight of a regulatory body, with variations in job titles (such as nurse aides, unregulated care providers, homecare workers), roles and responsibilities across settings and jurisdictions.^17,18^ These variations lead to difficulties estimating number of care workers based on examples from Norway, Australia and Canada.^19^ The US estimated there were 3,689 million home health and personal care aides making it the largest occupation in 2023^20^ but only a proportion of them delivered palliative and end-of-life care in the community. A recent workforce report estimated that 12,200 nurses and healthcare assistants were employed in hospices during 2022–2023.^21^ However, this report did not provide separate figures for nurses and healthcare assistants.^21^

The factors shaping wellbeing among this substantial yet under-researched workforce remain unclear. Workplace wellbeing is influenced by what people think and feel about their working lives, such as the quality of their relationships, resilience, sense of autonomy, the realisation of their potential, and their overall satisfaction with work.^22–24^ While previous studies have identified numerous factors that affect the wellbeing of healthcare workers, the focus has predominantly been on professional groups such as nurses and physicians.^9,12,25,26^ Some general stressors include organisational change, insufficient organisational support and workload.^27,28^ Hospice staff, however, face particular challenges involving emotional labour, given the frequent encounters with complex and sensitive issues relating to loss, death, and grief.^27,29–31^

For healthcare assistants providing palliative and end-of-life care in the community – providing care and support for people with palliative and end-of-life care needs – these challenges may be exacerbated by the nature of their work. Working alone in patients’ homes without other practitioners present, they are commonly referred to as ‘lone workers’.^32,33^ As such they may be required to make decisions in highly distressing situations,^32^ while often working at night. Healthcare assistants often have limited interactions with other professionals and team members,^34,35^ which restricts their opportunities for experiential learning and peer support.^32,36,37^

Despite these challenges, we previously demonstrated that healthcare assistants providing palliative and end-of-life care in the community report higher wellbeing scores (Warwick-Edinburgh Mental Well-being Scale mean = 52.2, standard deviation, SD = 8.6)^38^ compared to those in other healthcare settings^3,39,40^ and a general United Kingdom population,^41^ alongside low intention to leave scores (Turnover Intention Scale mean = 14.9, SD = 5.3, scale range 6–30).^38^ Moreover, we found that higher wellbeing scores were related to lower intentions to leave (*r*(216) = −0.25, *p* < 0.001), with workplace support emerging as a predictor of both outcomes.^38^ While providing end-of-life care is often seen as meaningful and professionally fulfilling,^10,42^ the wider factors contributing to healthcare assistants’ wellbeing and intention to leave are poorly understood. The aim of the present study was to examine these factors among lone working healthcare assistants providing palliative and end-of-life care in the community, offering a deeper understanding of what shapes these outcomes. Understanding the factors that contribute to wellbeing and intention to leave is essential for organisations seeking to improve staff retention and address workforce challenges.

## Methods

### Design

Qualitative study combining free-text responses from the cross-sectional survey, which provided breadth of data, with semi-structured interviews, which enabled in-depth exploration. Both datasets were analysed using reflexive thematic analysis.^43,44^ Such a design allowed for wide and in-depth exploration of factors affecting wellbeing and intention to leave among the study population. This study adopts a constructionist approach, recognising that healthcare assistants’ experiences and the meanings they attribute to their work are socially constructed through interactions within their organisational and care contexts. The study is reported following the Reflexive Thematic Analysis Reporting Guidelines (RTARG).^45^ This paper is part of a wider study. Our previously published work reported quantitative findings integrating qualitative findings specifically linked to healthcare assistants’ wellbeing support in the workplace.^38^ The current paper extends this to provide comprehensive qualitative exploration necessary to understand not just that wellbeing and intentions to leave vary, but why.

### Setting

Participants were drawn from a not-for-profit organisation in the United Kingdom providing care and support to over 60,000 terminally ill patients each year. The organisation’s hospice at home service employs healthcare assistants to deliver both scheduled (when healthcare assistant stays with one patient, mainly overnight) and emergency care in patients’ homes (multiple visits during a day or night).

### Participants and sampling

Eligible participants included healthcare assistants who spent any portion of their work hours providing palliative and end-of-life care working alone in patients’ homes. At the commencement of recruitment (01/05/23) 969 lone working healthcare assistants worked across the UK within hospice at home services, mainly during the night. We used convenience sampling to recruit survey participants. All who expressed interest in an interview (*n* = 14) were interviewed to maximise participation from this under-researched workforce.

### Recruitment

An invitation was distributed to all eligible healthcare assistants via email by the service managers. Two reminders were sent to enhance response rates, with survey participants entered into a prize draw and interviewees receiving a gift voucher. Completion of the anonymous survey was taken as an indication of consent to participate. The first question of the questionnaire acted as eligibility screening for lone workers. At the conclusion of the survey, participants could express their interest in an interview by completing a form via a separate link. Willing participants were contacted by KP via telephone or email to obtain consent and arrange interviews.

### Dataset generation

The survey was conducted using Microsoft Forms from 12/04/23 to 7/06/23 and included free-text questions:

1. What factors come to mind when you think of reasons not to stay in your current role?
2. What factors come to mind when you think of reasons to stay in your current role?
3. And finally, what do you think [the organisation] could do to support Health Care Assistants and help them stay in their role? That is, increase retention?

Full survey tool and data can be found in our previously published paper.^38^ All participants who filled an expression of interest form (*n* = 14), participated in the interviews. Semi-structured online interviews were conducted by KP between 19/07/23 and 31/08/23 using a topic guide (see Supplemental Material). Participants answered demographic and work characteristics questions at both stages. Dataset generation tools were developed by the research team with stakeholder advisory group input. The survey required no piloting; the first interview served as a pilot with no subsequent modifications needed.

### Data analysis

The free-text responses from the survey were organised using Microsoft Excel. Interview recordings were transcribed by a professional transcriber. All data have been anonymised. Both datasets were integrated throughout the analysis. We gave pseudonyms for interview participants using Anglo female and gender-neutral names to protect participants’ identities and reduce any cultural assumptions. Both datasets were then printed to facilitate pen-and-paper analysis. They were thoroughly read several times to ensure a deep understanding, with reflexive notes recorded throughout to track our assumptions and evolving interpretations.^46^ KP, who is a palliative care nurse within the same organisation, with experience of training, education and support of hospice at home staff, completed the analysis using reflexive thematic approach.^43,44^ Reflexive thematic analysis was selected because it aligns with our constructionist approach. This approach focussed on how participants constructed meaning through social interactions rather than seeking objective truths. Coding was primarily inductive, working at both semantic and latent levels to examine participants’ explicit accounts and underlying meanings within their organisational contexts. All data were independently coded by both KP and FH, who then met to compare and discuss their analyses until consensus was reached on codes and themes. All findings were subsequently discussed with the wider research team and stakeholder advisory group, and refined based on their feedback and insights (please see authorship section for more details).

### Ethical considerations

Ethical approval was received from Ulster University Nursing and Health Sciences Ethics Filter Committee (FCNUR-23-010; 08/02/23) and the Senior Research Governance Manager at the recruiting organisation (31/03/23). The study was conducted in accordance with ethical principles for research involving human participants. No ethical issues arose during the conduct of the study.

## Results

### Overview

Response rate for the survey was 22.5%. One hundred and eighty-three out of 218 survey participants completed optional free-text questions. This was followed by 14 semi-structured interviews ranging from 24 to 67 min (mean of 41 min). Participant characteristics are described in Table 1.

**Table 1.** Sample characteristics.

Variable	Category	Survey participants, *n* = 218		Interview participants, *n* = 14	
		Frequency^a^	% of valid responses/mean (SD)	Frequency	% of valid responses/mean (SD)
Gender^b^	Female	202	92.7	14	100
	Male	9	4.1	-	
Age		200	Mean 51.3 (SD 11.0)	14	Mean 51.2 (SD 13.9)
Ethnicity^c^	White	181	85.0	14	100
	Ethnic minorities	32	15.0	-	-
Proportion spent lone working	All/almost all	165	75.7	14	100
	Most	23	10.6	-	-
	About half	7	3.2	-	-
	Some	16	7.3	-	-
	Almost none	7	3.2	-	-
Contract	Contracted hours	169	77.9	10	71
	Bank	48	22.1	4	29
Type of hours	Full time	83	39.3	2	14
	Part time	128	60.7	12	86
Place of work	England	144	68.6	8	57
	Northern Ireland	18	8.6	2	14
	Scotland	39	18.6	4	29
	Wales	9	4.3	-	-
Total years of experience as healthcare assistant^d^		199	Mean 18.7 (SD 11.7)	14	Mean 14.4 (SD 12.0)

SD: standard deviation.

a Missing data: gender (seven participants, 3.2%), age (18, 8.2%), ethnicity (5, 2.3%), type of hours (7, 3.2%), place of work (8, 3.7%), years of experience as healthcare assistants in current employing organisation and total (19, 8.7%), overall level of missingness 10.6%.

b Includes survey responses: prefer not to say (4), prefer to self-describe (2) and missing (1).

c Ethnic minorities included healthcare assistants self-reporting as Asian (3), Black (23) and other/mixed categories (6).

d Missing includes survey participants whose years of experience exceeded their age or required commencing work before age 16.

### Factors impacting on wellbeing and intention to leave

Our analysis generated several factors impacting healthcare assistants’ wellbeing and intention to leave and grouped these into three levels: societal, organisational and individual.

#### Societal-level factors influencing wellbeing and intention to leave

This theme encapsulates participants’ thoughts and feelings about how society and others perceive their role and contribution. These perceptions span a wide spectrum. On one end, healthcare assistants report that they feel invisible and undervalued within wider society, while also recognising that people affected by terminal illness highly value their contribution.

**Public perceptions of the healthcare assistant’s role** are marked by a lack of understanding regarding the complexity and responsibilities involved, further reflected by low pay across the care sector. Participants shared being described as ‘sitters’, which they find demeaning and reductive of their actual contributions.

*I think it would be beneficial to have more education publicly about the role of the healthcare assistant, and to push for more knowledge round that, so that there is more respect within the community.*

*(Harper, interview participant)*

Mixed experiences related to **value and recognition of their role** were reported when working with other healthcare professionals, such as district nurses. Some healthcare assistants reported that a lack of acknowledgement in contributing to information or decisions around patient care impacts their sense of professional worth.

*Sometimes what you tell them [other healthcare professionals] isn’t taken any notice of, and you just feel as though you don’t know anything and you’re telling them something. I don’t think we get valued.*

*(Anne, interview participant)*

**Positive verbal feedback** from patients and their families greatly enhances job satisfaction and confidence, highlighting the meaningfulness of their work. Such feedback brings a sense of value and validates healthcare assistants’ efforts to provide the best possible care.

*I like it whenever people say, we really appreciate what you did for our mum or our dad. Or we really appreciate the chance that we had to chat through things with you and maybe learn a few new things ourselves about how to look after them. We are glad that you were here last night to deal with that situation because it was quite tough. It makes me want to keep doing the job!*

*(Lisa, interview participant)*

#### Organisational-level factors that influence wellbeing and intention to leave

This theme contains factors relating to work organisation, management practices, and workplace support that fall within the remit of the employing organisation to influence. Central to this theme is the tension between autonomy and isolation inherent to lone working and healthcare assistants’ need for meaningful connection, support and recognition within their workplace.

**Lone working** poses challenges, including unpredictability and the responsibility of managing situations independently as the sole practitioner present in the home. While this is recognised as an inherent aspect of the role, participants emphasised the importance of knowing whom to contact for assistance and having access to up-to-date patient information prior to their shifts to reduce uncertainty.

*You get concerned that you’re going to have a situation that you’re not necessarily sure how to deal with. Because the patients change quickly. You don’t necessarily know what’s going to happen. . . obviously you are in someone’s house and you are by yourself. . . because you are there on your own, you are having to make those decisions.*

*(Sam, interview participant)*

Furthermore, healthcare assistants who mainly work at night are required to undertake **unpaid work-related tasks** outside of shift hours, such as calling their line manager and contacting IT support. Frequent organisational and system changes require them to spend time troubleshooting and learning new systems, often without adequate support or training, leading to frustration, which may influence staff turnover.

*That would be one of the things why I think people probably leave, because there is so much pressure to do so much outside of work*

*(Amber, interview participant)*

**Workplace culture** should involve recognising employees as individuals with unique social and emotional needs and providing mechanisms to address these within the workplace. Participants emphasised the importance of interactions with **peers** to fulfil these needs, describing colleagues as uniquely placed to provide emotional support because of shared experiences.

*I much prefer to talk to my colleagues. Because like I said, they’ve been in those situations so they kind of know what you’re going through. (. . .) With your colleagues, you can have a cup of coffee, you can laugh, you can joke, you can cry.*

*(Morgan, interview participant)*

A safe and trusting relationship with the **line manager** is crucial for healthcare assistants, as they serve as the primary link between lone workers and the organisation. Line managers’ multifaceted role includes addressing wellbeing, providing performance feedback, facilitating debriefs, and fostering team cohesion and a supportive workplace culture.

*They’ve always been really good. They’ll help change your shifts. They’ll be a listening ear if you’ve got an emotional problem. They can guide you to the help that is out there.*

*(Sonia, interview participant)*

**Inadequate pay** emerged as a significant factor affecting both wellbeing and intention to leave. Participants expressed concerns that their remuneration does not adequately reflect the complexity of their responsibilities, their experience or training. While stability of the regular income is appreciated, it is often viewed as insufficient given the demanding nature of their role, the emotional labour involved, and current market conditions.

*Pay - not a true reflection of the care we provide to not only patients but families as well and due to the living crisis it does have its toll and effect on you both mentally and physically. we really are not paid enough for what we do and the effect it has on our emotional wellbeing.*

*(Survey participant 124)*

**Training and career progression opportunities** are important for personal development and confidence. Participants shared a need for more frequent and accessible learning opportunities and additional responsibilities to better utilise their skills.

*Wanting to improve myself been offered more responsibility and acknowledge for my experience I bring from previous jobs and been rewarded for that. as well as opportunity to do further training for self-improvement and development to keep the fire burning inside for the passion I have for helping others and families and to be able feel more involved and not just a HCA [healthcare assistant] and the feeling of been restricted in my role.*

*(Survey participant 106)*

However, participants reported a gap for individuals who want to progress within their current role rather than transition into management, nursing or non-clinical positions.

*I personally don’t believe, in my role, I could go any higher. I don’t think there’s any progression higher, unless I left there and went into university and started doing nursing or something. (. . .) Unless I came out of the healthcare assistant into something else.*

*(Emilia, interview participant)*

#### Individual-level factors associated with wellbeing and intention to leave

The final theme focuses on individual factors. While universally healthcare assistants gain significant job satisfaction from the meaningful nature of their work, challenges such as the emotional demands of caregiving and lone working affect individuals differently based on their personal circumstances, qualities, skills and experiences.

The vast majority of healthcare assistants agreed in their responses that they stay in their role due to profound **job satisfaction and the sense of meaningfulness** they feel when able to help people impacted by terminal illness. This has been the strongest finding from the free-text data.

*My passion is end of life care and I want to provide the best care I can with professionalism and dignity. Knowing that I can make a difference to the patient and to the families is what keeps me going. When families tell me that I made a difference then that’s my reward*

*(Survey participant 16)*

Such sentiment was shared by interviewees who described a sense of deep **internal motivation to help others** and a reward when they can bring others some relief during difficult times.

*I love my job. Absolutely love my job. I love supporting families, helping them. I’ve been in this situation. I know what it’s like to be looking after somebody. (. . .) I’m not saying it’s not sometimes sad. And some patients are much harder than others, depending on the family dynamics. But certainly, I think it’s a real rewarding job. And I think you come away feeling you’ve really achieved something.*

*(Chris, interview participant)*

However, this commitment to care suggests that organisations may be relying on healthcare assistants’ dedication to compensate for systemic shortfalls.

*We all put in so many extra hours without pay and you will never hear anyone complain about that. We all put our patients and their families first.*

*(Survey participant 178)*

The overall sense of meaning and job satisfaction appears to buffer healthcare assistants from negative emotional impact of caring for people affected by terminal illness.

*It can be very upsetting, but that comes as part of the job. Because I always say, if you can’t cry, it’s not for you. Because we all have emotions and everything. (. . .) I do my crying in the car. That’s where I have my emotions, when I leave. (. . .) And then I think, well . . . I’ve been there for them and they’ve been looked after well. And I know that most of them have had a good death.*

*(Julie, interview participant)*

**Personal qualities and skills** may affect how healthcare assistants experience the demands of the role. Lone workers who thrive in hospice at home settings typically exhibit confidence, independence, adaptability, and a positive attitude towards challenges. However, lone working is not suitable for everyone; while some adapt well to the responsibility and autonomy that comes from working independently, others may find the isolation and pressure of making decisions with limited support detrimental to their wellbeing. Those who had more experience as lone workers appeared to be coping better with such demands.

*You need to be a certain kind of someone that can walk into a house, not knowing anybody, not knowing the layout. . . you really have to get in, and you have to be able to adapt very easily and very quickly, to different situations.*

*(Val, interview participant)*

Some participants reported that night working significantly impacts **physical health**, leading to disturbed sleep patterns and ongoing fatigue and as a result, they may need to reduce their hours or find alternative employment.

*Constant tiredness as only night shifts are available, that is the nature of the job. I am aware that nightshifts are not sustainable nor healthy for the rest of my working life and so eventually I will need to reduce my shifts and find work elsewhere.*

*(Survey participant 83)*

## Discussion

This study reveals key insights into the experiences of lone working healthcare assistants providing palliative and end-of-life care in the community. Healthcare assistants experience a fundamental paradox - high job satisfaction coexists with the perception that their role is undervalued both financially and in terms of their skills and contributions to society. Secondly, many of the factors negatively impacting their wellbeing and retention intentions fall within organisational control, providing opportunities for targeted interventions.

### The paradox of meaningful work and undervaluation

Job satisfaction and meaningful work emerged as the main reasona for healthcare assistants to remain in their roles, despite multiple factors negatively impacting their wellbeing. This aligns with other research on hospice workers^42,47,48^ and homecare workers,^10,49,50^ where supporting people at end-of-life contributes to psychological wellbeing. Evidence suggests job satisfaction among homecare workers may be linked with close relationships developed with patients and family caregivers,^10,51^ and the sense of being able to ‘make a difference’.^42^

This meaningful work exists alongside systemic undervaluation reflected in low societal status^10,52–54^ and exclusion from patient care decisions despite providing most direct care.^55–57^ Evidence shows that over time healthcare assistants have increasingly taken on roles traditionally performed by nurses, yet this shift has not been matched by appropriate recognition or support.^58,59^ While the sense of doing meaningful work acts as a strong predictor of retention,^60^ it may mask underlying sustainability issues related to systemic undervaluation that could pose long-term retention risks.

This situation raises important questions about the sustainability of relying on intrinsic motivation to compensate for systemic undervaluation. While meaningful work provides psychological protection, it may also enable organisations to maintain suboptimal working conditions, assuming that job satisfaction will overcome structural challenges. This risks exploiting healthcare assistants’ commitment to caring.

### Organisational factors as opportunities to make positive impacts

Lone working practices appear to have negative impacts on wellbeing, highlighting the need for organisations to carefully structure and support these arrangements. Previous research has shown the challenges of delivering end-of-life care at home as a lone worker, with the unpredictability and rapidly changing nature of homecare situations further compounding these difficulties.^32,37,61^ Healthcare assistants in our study emphasised the importance of establishing trusting relationships with line managers, who serve as their primary organisational connection, to cope with these challenges, similarly to general homecare workers.^62^ Literature highlights that strong organisational support and supportive culture within a workplace, such as supervisor support and peer support, could improve hospice staff wellbeing, despite this, such support is often inadequately available.^10,34,36,42,52,63^ This is surprising given the emotional labour involved in their role.^32,52,64^ Healthcare assistants often develop close relationships with the people they support through sharing the profound experience of accompanying someone through dying, and may consequently experience grief.^62,65,66^ Supportive workplace culture experienced by some participants in our study may help to explain high levels of wellbeing described in our previous paper.^38^ For others who lack such support, interventions are needed to manage these emotional challenges.

While societal-level recognition is difficult to influence, organisations can directly impact how healthcare assistants feel valued. This could be done by ensuring pay levels reflect their responsibilities, demonstrating organisational recognition of their role’s complexity and market conditions.^59,67,68^ Secondly, provision of appropriate training and career progression opportunities signals investment in their professional development. A review by Papworth et al.^42^ indicated a strong relationship between appropriate training and wellbeing in hospice staff, yet training provision remains insufficient and patchy.^69^ Previous research demonstrated that a perceived lack of career advancement opportunities significantly increases turnover intention among homecare workers.^60^ It is worth noting that healthcare assistants want to be valued for doing their current job well rather than being pressured towards progression into nursing or management roles,^56^ and career progression opportunities should reflect this.

Unlike other home healthcare settings, participants did not report traditional occupational hazards as primary concerns, possibly reflecting the protective effects of meaningful work or existing safety systems.^70–73^

### Considerations for policy and practice

**Figure 1.**
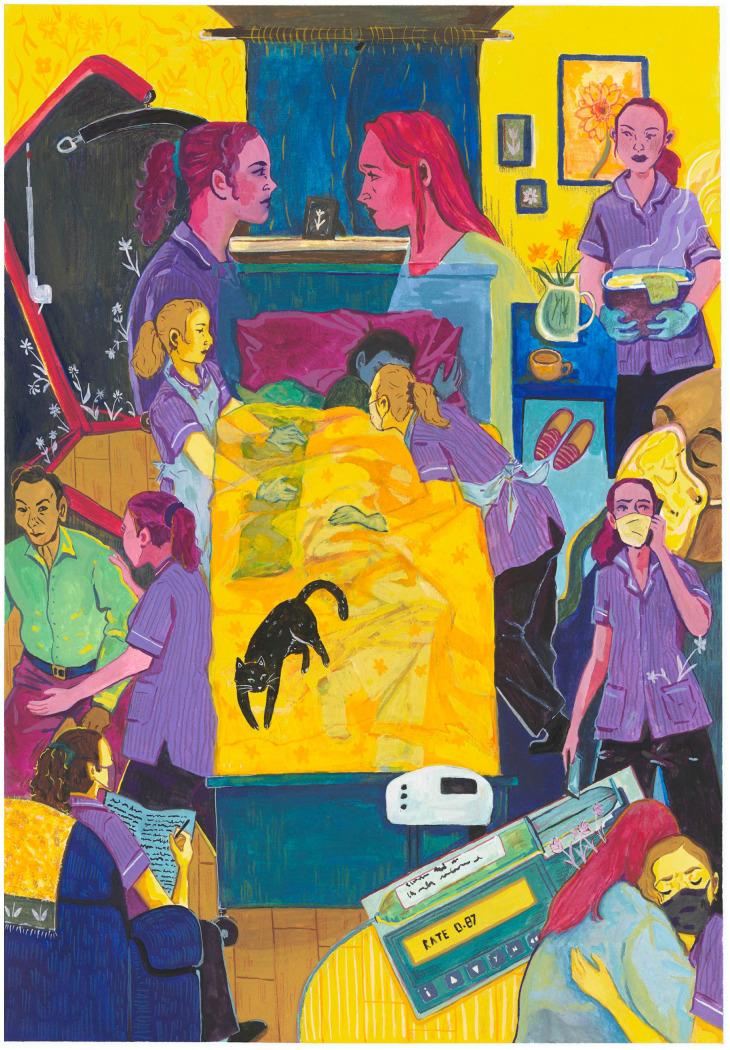
Painting by Dáire Lawlor, Northern Ireland-based artist and healthcare assistant, depicting the multifaceted role of healthcare assistants providing palliative and end-of-life care in the community. This artwork was commissioned by the project lead KP and funded by study grant MCSGS-22-801.

Organisations should prioritise investment in workplace culture fostering team interaction and trusting line manager relationships. Recognition systems must reflect role complexity through appropriate pay and career progression opportunities that value current role performance rather than suggesting transition to nursing or management. Finally, hospice organisations can influence perceptions of the role through community education and by ensuring healthcare assistants are visible in public-facing activities, moving beyond their invisible status in healthcare hierarchies. To support this, the research team commissioned artwork by a practising healthcare assistant (Figure 1), which captures the emotional demands, clinical judgement, and multifaceted interactions that define their work – yet remain invisible to healthcare systems and society.

### Implications for research

Future research should test organisational interventions, particularly peer support programmes and mentorship schemes. Longitudinal studies tracking career trajectories and economic evaluations of wellbeing investments are needed to explore sustainability of lone working arrangements and implications for investing in staff wellbeing.

### Strengths and limitations of the study

This study addresses a significant gap in palliative care research by focussing on healthcare assistants. The strengths include an approach combining qualitative survey responses with in-depth interviews and stakeholder involvement enhancing practical relevance. Limitations include convenience sampling from a single charitable organisation, where participants worked mostly providing scheduled overnight care, predominantly female and white sample, cross-sectional design, and inability to establish causal relationships between wellbeing and intention to leave. We acknowledge that KP’s position as a nurse researcher within the employing organisation shaped the research process and interpretation. This insider position provided contextual understanding but required ongoing reflexivity about assumptions and the risk of over-identifying with organisational perspectives. The involvement of FH as an external researcher provided critical distance, and collaboration between insider and outsider perspectives strengthened analytical rigour.

## Conclusion

This study reveals that lone working healthcare assistants providing palliative and end-of-life care in the community experience high job satisfaction from meaningful work alongside systemic undervaluation. Healthcare assistants perform complex, autonomous work in emotionally demanding environments, yet this complexity remains largely invisible. For healthcare systems increasingly reliant on healthcare assistants for home-based care delivery, addressing this paradox is essential for sustainable service provision. The clear identification of organisational-level intervention opportunities provides a foundation for targeted improvements, while recognising healthcare assistants’ complex work challenges assumptions about skill requirements and support systems in community healthcare.

## Supplemental Material

sj-docx-1-pmj-10.1177_02692163261426184 – Supplemental material for ‘You’re a human being and not a robot that goes out to work’: A qualitative study exploring factors impacting on wellbeing and intention to leave among lone working healthcare assistants providing palliative and end-of-life care in the community Supplemental material, sj-docx-1-pmj-10.1177_02692163261426184 for ‘You’re a human being and not a robot that goes out to work’: A qualitative study exploring factors impacting on wellbeing and intention to leave among lone working healthcare assistants providing palliative and end-of-life care in the community by Katarzyna A. Patynowska, Tracey McConnell, Anne Finucane, Emma Maun, Erin Raquel Fantoni, Jonathan Clemo, Epiphany Leone, Natasha Wynne and Felicity Hasson in Palliative Medicine
