# Supplementary material for: ‘You’re a human being and not a robot that goes out to work’: A qualitative study exploring factors impacting on wellbeing and intention to leave among lone working healthcare assistants providing palliative and end-of-life care in the community
Source: Palliat Med. 2026 Mar 18;40(6):833–44. doi: 10.1177/02692163261426184 (PMC13221572; doi:10.1177/02692163261426184)
Supplement: sj-docx-1-pmj-10.1177_02692163261426184 – Supplemental material for ‘You’re a human being and not a robot that goes out to work’: A qualitative study exploring factors impacting on wellbeing and intention to leave among lone working healthcare assistants providing palliative and end-of-life care in  [file sj-docx-1-pmj-10.1177_02692163261426184.docx]

**Supplemental material for paper:**

***‘‘you're a human being and not a robot that goes out to work’:***

**A qualitative study exploring factors impacting on wellbeing and intention to leave among lone working Healthcare Assistants providing palliative and end-of-life care in the community.**

**Patynowska K, et al. (2025)**

# **Interview schedule**

Opening Statements

The researcher will start with an opening statement as follows:

- *Thank you for taking time for this interview today, it* will last between 30-60 minutes *and can be stopped at any time*.
- *I want to remind you that there are no right, or wrong answers. You are asked not to disclose any specific patient cases or identifying features in your responses. As per the PIS/Consent form all* *disclosed information will be treated confidentially unless required by law, i.e. there is a risk to yourself or others.*
- *Our questions today have been informed by the survey that was sent out to all community based lone working HCAs in Marie Curie, in terms of what issues to focus on. Please keep in mind that all the questions are focusing on your perspective of HCAs work-related psychological wellbeing, support needs and staff retention.*

The researcher will seek verbal consent for digital recording (video and audio), otherwise notes will be taken

Opening questions

- Can you outline how long you have been working as a Healthcare Assistants in Marie Curie Community Service?
- What is your previous work experience?
- In what services do you usually work and what hours do you do?
- How much lone working do you do in a typical month?
- How much do you work within clinical working hours (8am-6pm) and during the out-of-hours working period (6pm-8am)?

1. Thinking about your role as HCA, can you describe your **work-life balance**? How does your as HCA impact your work-life balance? Does it have any impact on your intention to stay, or leave their job? In what way?
2. What type of **training, development and career progression opportunities** in Marie Curie are available to you? Does it have any impact on your work-related wellbeing? In what way? Do you think these also impact on your intention to stay, or leave their job? In what way?
3. How important is **feeling valued** to you in your role in terms of your psychological wellbeing at work? Could you tell me a little more about why you think that? In what way does it impact on your intention to stay, or leave their job?
4. What does **working as HCA mean to you**? Do you think your role as a HCA has a positive **impact on the lives** of patients/families? In what way? What impact does it have on your wellbeing at work? What impact does it have on your intention to stay in/leave your job?
5. How **contact with other HCAs, healthcare practitioners and line manager** impact on your wellbeing? How would you describe the support from these sources? How that support looks like and what does it incorporate? What impact do these have on your intention to stay in/leave your job?
6. What is **the role of line manager in the support** offered to HCAs? *(explore the line manager’s role in terms of providing the support, facilitating support e.g. team meetings and signposting to support)*
7. Very few people who completed the survey heard and/or used **Employee Assistance Programme, Wellbeing Hub, Schwartz rounds** – have you heard of these? What do you think are the reasons for low awareness and usage of those support systems? *(explore differences between formal vs. informal or person to person vs. virtual support)*
8. How do you think people **choose the sources of support** they access? *(explore if there flexibility in accessing various support systems or people tend to use the same sources; are people use various sources of support for ‘wellbeing at work’ vs. ‘wellbeing as a person’)*
9. What support has been **most and least helpful to you** at work? In what situations did you use it? *(explore appropriateness of support accessed for specific issues, i.e. was support accessed the most appropriate in a specific situations)*
10. **Have you considered leaving** your current role? Can you tell me more about that, please? *(explore the main considerations for staying and leaving)*
11. What **support intervention do you think has the most potential** to help improve the wellbeing, job satisfaction and retention of HCAs? Why did you choose that particular one?
12. What would your **second choice** be? Why did you choose that particular one? What would be your second choice of the intervention?

Final question

Is there something else you would like to add before we finish?

End of interview

Thank you very much for your time and for sharing your thoughts

Signpost to PIS support section
